# A Functional Polymorphism (rs937283) in the MDM2 Promoter Region is Associated with Poor Prognosis of Retinoblastoma in Chinese Han Population

**DOI:** 10.1038/srep31240

**Published:** 2016-08-10

**Authors:** Yongfa Jiao, Zhongming Jiang, Yuxia Wu, Xiaochong Chen, Xing Xiao, Haiying Yu

**Affiliations:** 1 Department of Ophthalmology, Yishui Central Hospital, Linyi, Shandong, China; 2 Yishui Center for Disease Control and Prevention, Linyi, Shandong, China; 3 Department of Ophthalmology, Shandong Provincial Qianfoshan Hospital, Jinan, Shandong, China; 4 Department of Orthopedics, Shandong Provincial Qianfoshan Hospital, Jinan, Shandong, China; 5 Department of Radiology, Shandong Cancer Hospital and Institute, Affiliated to Shandong Academy of Medical Science, Jinan, Shandong, China

## Abstract

The effect of single nucleotide polymorphisms (SNPs) at MDM2 has been investigated in several cancer types. Three MDM2 SNPs(rs937283, rs2270744 and rs769412) have previously been suggested to be positively correlated with cancer. In this study, we aimed to explore the association of rs937283, rs2270744 and rs769412 polymorphisms with retinoblastoma (RB) risk, clinicopathological characteristics, and prognosis. Compared with wild-type genotype AA at rs937283, individuals carrying AG and GG genotype had a significantly increased risk for developing RB (OR = 1.86, 95% CI 1.13–3.08; OR = 2.48, 95% CI 1.10–5.62, respectively). RB patients with allele G at rs937283 were more susceptible to invasion and high tumor aggression (OR = 2.42, 95% CI 1.43–4.11; OR = 2.15, 95% CI 1.27–3.64, respectively). Kaplan-Meier curves and log-rank results revealed that RB patients harboring genotype GG and G allele at rs937283 had worse survival (*P* < 0.02 and *P* < 0.01, respectively). In addition, the A to G substitution at rs937283 significantly enhanced the transcription activity of the MDM2 gene *in vitro. In vivo*, we found that MDM2 mRNA and protein were overexpressed in individuals who carried the G allele at rs937283. This study suggested that the MDM2 rs937283 polymorphism is a novel functional SNP both *in vitro* and *in vivo* as well as a biomarker for poor prognosis in RB.

With an estimated incidence between 1 in 16,000 and 1 in 20,000 live births, retinoblastoma (RB) is the most frequent intraocular malignancy among children worldwide^1^,^2^. RB has a profound effect on infants’ quality of life and it is estimated that approximately 9,000 newly-diagnosed pediatric patients will die every year^3^. The development of RB may be heritable and non-heritable, which are respectively associated with germline and somatic mutations in RB1 tumor suppressor gene^4^. As a result of the expansion in knowledge underlying RB etiology, improvement of public and medical awareness, and development of rigorously innovative clinical treatment, RB is no longer considered a deadly childhood disease^5^. However, the survival rates vary widely around the world, mortality from RB is approximately 70% in countries of low and middle incomes. Because improving overall survival and vision depends on the severity of disease at presentation, early diagnosis and accurate prognosis evaluation is necessary. Thus, recent investigations have focused on the identification of RB genetic biomarkers such as gene polymorphism, lnRNA, and microRNA, which can affect disease progression, and effectively deepen our understanding of RB pathogenesis, as well as serve as potential molecular prognostic indicators that ultimately lead to the development of novel therapeutic strategies^6^,^7^,^8^.

Other than the *RB* gene, studies have shown that a number of other genes, including *p21, p53, MDM2,* and *MDM4*, may influence the development of RB^9^. p53 pathway is the master control system of cell cycle, genome stability, and cell apoptosis, which could be modulated by a negative feedback loop in which p53 could transcriptionally activate MDM2 (murine double-minute 2 homology), which in turn could lead to increased proteolytic degradation of p53, thus MDM2 functions as a negative regulator of p53 pathway^10^. Furthermore, MDM2 could interact with pRB and bind to the activation domain of E2F1 transcription factor which could inhibit pRB regulatory function^11^. Thus, MDM2 was identified as a modifier gene in RB^12^. Recent data suggested that MDM2 gene polymorphisms may contribute to increased MDM2 basal expression and increase cancer susceptibility. Specifically, MDM2 rs2279744 (SNP T309G) polymorphism has been shown to produce a higher-affinity DNA-binding site for Sp1, which could increase MDM2 mRNA and protein expression, and thus increase the degradation of p53 and hinder p53-induced apoptosis^13^. Increased MDM2 expression is related to elevated cancer risk in sporadic and hereditary malignancies and increased likelihood of distant metastases^14^. Previous studies have found a positive association between MDM2 rs2279744 polymorphism and RB development^9^,^15^. In addition to rs2279744, recent studies have suggested the potential association of rs769412 and rs937283 in the MDM2 loci with cancer development^16^,^17^. rs769412 polymorphism (also known as G354A) is a A to G substitution located at the 354 nucleotide in the exon 12 of MDM2 gene, while rs937283 (also known as G2164A) polymorphism could lead to an A to G base change at the 2164 nucleotide in the promoter region of MDM2 gene^18^,^19^. Previous studies have reported rs769412 and rs937283 polymorphism were associated with the development of several cancer types^16^,^18^. Until now, there has been no report investigating the association of these two polymorphisms with RB development. To determine the role of MDM2 polymorphism in RB, we analyzed the distribution of rs937283, rs2270744 and rs769412, and assessed the association of these polymorphisms with clinicopathological characteristics and prognosis in Chinese RB patients. Further, we conducted the molecular work to confirm whether the variants could alter MDM2 expression.

## Results

### Participant characteristics

A study on 137 RB patients and a control group of 150 non-carriers of RB was undertaken. All participants were Han ethnicity from the same region in Shandong of China and there was no statistical difference in gender between RB patients and normal controls (*P* = 0.47). The clinical characteristics of RB patients were presented in Table 1.

### Association of rs937283 with RB risk

As shown in Table 2, significant differences in the distribution of rs937283 were observed between RB patients and normal controls. The analysis did not yield a significant deviation from HWE in control group (*P* = 0.196). Compared with WT genotype AA at rs937283, individuals carrying genotype AG and GG had significantly increased risk for developing RB (OR = 1.86, 95% CI 1.13–3.08; OR = 2.48, 95% CI 1.10–5.62, respectively). Significantly increased risk for RB was also observed in subjects with genotype AG or GG under a recessive model (OR = 1.98, 95% CI 1.23–3.17). In addition, individuals with G allele at rs937283 had an almost 74% increased risk of RB development when compared with A allele (OR = 1.74, 95% CI 1.21–2.51).

### Association of rs2279744 with RB risk

The distribution and statistical analyses of rs2279744 genotype in RB patients and normal controls are also described in Table 2. The value for the χ2 tests of HWE was 0.777 for controls. Similar to rs2279744 polymorphism, we did not observe a positive association between rs2279744 polymorphism and RB risk. Although the frequency of TT genotype and T allele was a little bit higher in RB patients, there was no statistically significant difference (OR = 1.14, 95% CI 0.57–1.57; OR = 1.06, 95% CI 0.77–1.48; respectively).

### Association of rs769412 with RB risk

The distribution and statistical analyses of rs769412 genotype in RB patients and normal controls are summarized in Table 2. The value for the χ2 tests of HWE was 0.100 for controls. There is no statistically significant difference in the frequency of rs769412 between RB patients and controls. The frequency of GG genotype at rs769412 was increased in RB patients than controls when compared with WT genotype AA, but with no significant difference (OR = 2.02, 95% CI 0.70–5.84). Similarly, compared with allele A, no significant difference in the likelihood of RB development could be observed between individuals with allele A and G at rs769412 (OR = 1.31, 95% CI 0.89–1.93).

### Linkage Disequilibrium(LD) analysis

**Figure 1:**
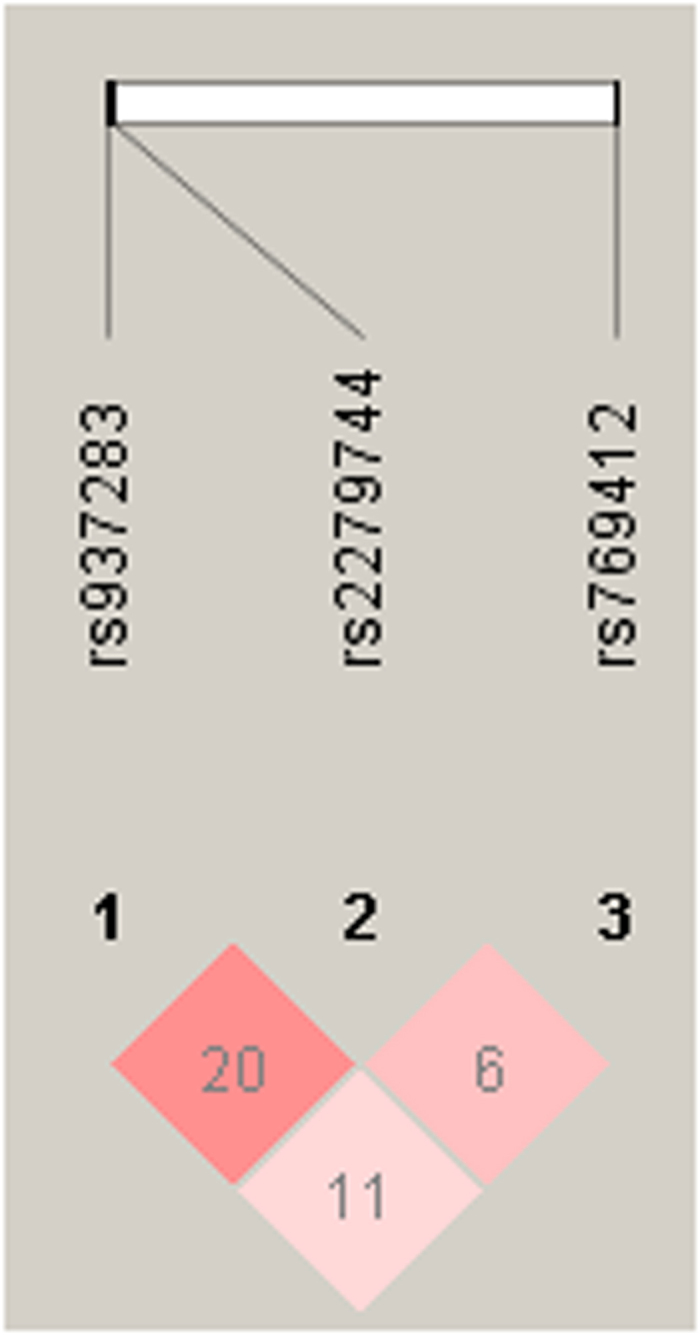
Linkage disequilibrium (LD) of rs937283, rs2279744 and rs769412 performed by Haploview 4.2 software in RB patients and normal control included in the present study. LD measure of r^2^.

Linkage disequilibrium of rs937283, rs2279744 and rs769412 were analyzed using Haploview 4.2 software (Fig. 1). The result revealed that these three SNPs were not in LD (r^2^ = 0.20, r^2^ = 0.11, r^2^ = 0.06; respectively).

### Association of rs937283, rs2279744 and rs769412 with clinicopathological characteristics in RB patients

The association of rs937283, rs2279744 and rs769412 with clinicopathological features in RB patients was further evaluated. The results of stratification analysis with parameters of gender, age at diagnosis, family history of RB, laterality, tumor invasion, tumor aggression, lag time were presented in Table 3. Although the association of rs769412 and RB risk is not positive, RB patients carrying AG genotype at rs769412 had a significant higher risk of tumor invasion (OR = 2.75, 95% CI 1.12–6.77). The results also showed that RB patients carrying allele G at rs769412 had a much higher risk for invasion (OR = 2.65, 95% CI 1.31–5.36). On the other hand, a positive association of rs937283 with invasion was also observed in RB patients with genotype AG or GG compared to WT AA genotype (OR = 2.73, 95% CI 1.30–5.75; OR = 4.90, 95% CI 1.44–16.6, respectively). In addition, RB patients with AG and GG were likely to have highly aggressive tumors (OR = 2.75, 95% CI 1.30–5.81; OR = 3.40, 95% CI 1.08–10.7, respectively). At the allele level, RB patients with allele G at rs937283 were more susceptible to invasion and high tumor aggression (OR = 2.42, 95% CI 1.43–4.11; OR = 2.15, 95% CI 1.27–3.64, respectively). However, no significant association of rs2279744 with clinicopathological characteristics could be identified.

### Association of rs937283, rs2279744 and rs769412 with RB prognosis

**Figure 2:**
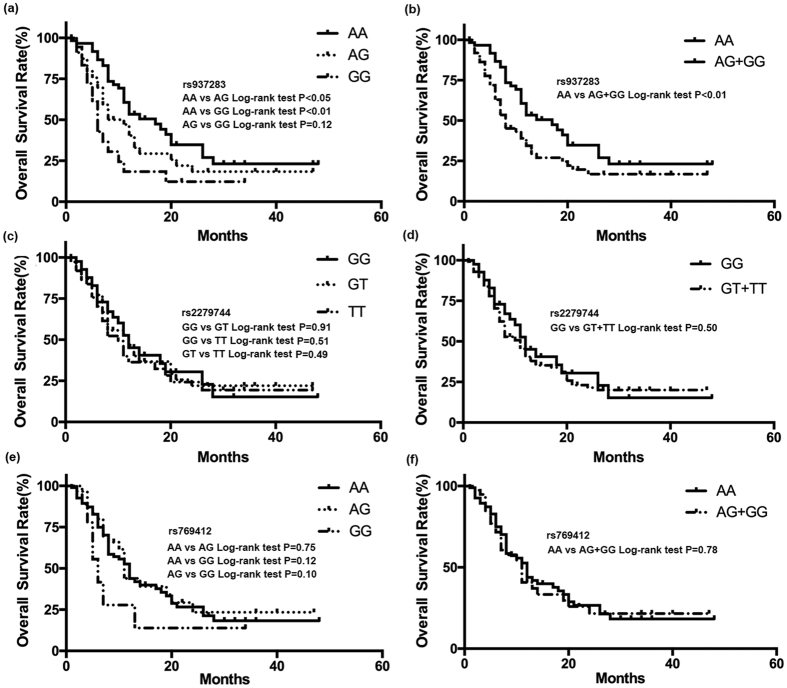
Kaplan-Meir survival curves. Kaplan-Meir survival curves of RB patients with different MDM2 rs937283 ((**a**,**b**) 33 events for AA, 34 for AG, 15 for GG;), rs2279744 ((**c**,**d**) 24 events for GG, 32 for GT, 26 for TT;), rs769412 ((**e**,**f**) 56 events for AA, 19 for AG, 7 for GG;) polymorphisms; MDM2 rs937283 polymorphism was correlated with the overall survival in RB patients (**a**,**b**), but not rs2279744 (**c**,**d**) and rs769412 (**e**,**f**).

Kaplan–Meier curves were constructed to evaluate the association of survival rate with rs937283, rs2279744 and rs769412 SNPs. Significant difference in survival rate was detected among patients with different genotypes at rs937283, but not rs769412 or rs2279744 (Fig. 2). Kaplan-Meier curves and log-rank results revealed that RB patients carrying genotype GG wat rs937283 had shorter survival time than those with genotype AA and AG alone (Fig. 2a, *P* < 0.05, *P* < 0.02, respectively). Consistently, RB patients carrying G allele (AG + GG) at rs937283 also had worse survival (Fig. 2b). In addition, the results of the multivariate analysis of survival time using the Cox proportional hazards model are presented in Table 4. Tumor invasion and lag-time were two independent risk factors for poor overall survival in RB patients (HR = 3.17, 95% CI 1.28–4.66; HR = 3.87, 95% CI 1.72–5.52). However, rs937283, but not rs769412 or rs2279744, was independently associated with overall survival. Genotype AG + GG carriers of rs937283 exhibited a significantly increased risk of poor overall survival *versus* GG genotype (HR = 2.01, 95% CI 1.04–3.95), suggesting that AG + GG genotype was an independent risk factor for overall survival in RB patients.

### Effects of rs937283 polymorphism on transcriptional activity

**Figure 3:**
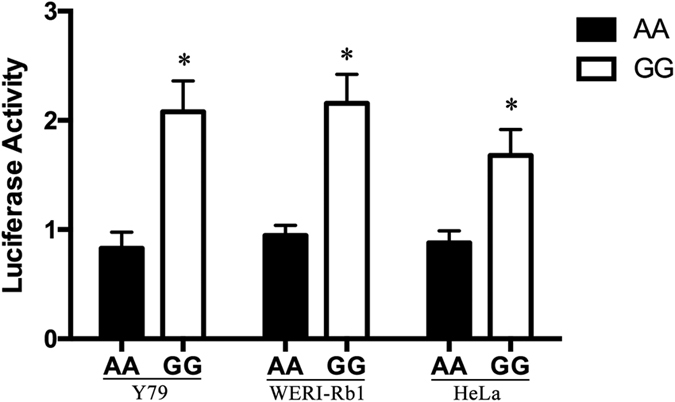
Effect of MDM2 rs937283 polymorphism in the MDM2 promoter activity. Schematic representation of reporter plasmids containing A or G allele at rs937283, which was inserted into upstream of the luciferase reporter gene in the pGL3 Basic plasmid. pRL-SV40 were cotransfected into Y79, WERI-Rb1, HeLa cells as the internal control of Renilla luciferase. Columns, mean from three independent experiments; bars, standard deviation. *P < 0.05 compared with the construct counterpart.

To evaluate whether the promoter activity could be affected by rs937283 polymorphism, we constructed luciferase reporter vectors (pGL3) with either A or G allele and used them for transient transfections with Y79, WERI-Rb1 and HeLa cells as mentioned in method section. Compared to those with A allele, the vectors with rs937283 G allele yielded a 90% to 150% increase in the relative luciferase activities in all three types of cell lines (*P* < 0.05 for all, Fig. 3). These results suggested that rs937283 G allele indeed could increase transcriptional activity of the MDM2 gene *in vitro*.

### Association of rs937283 polymorphism with mRNA and protein expression levels of MDM2

**Figure 4:**
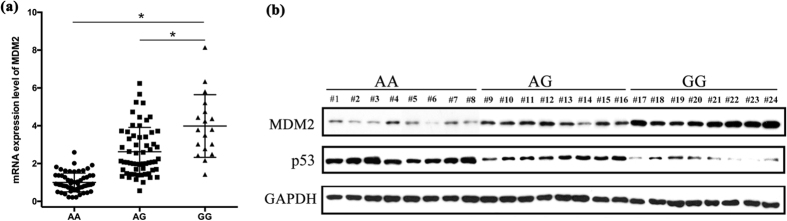
Association between rs937283 polymorphism and MDM2, p53 expression. (**a**) MDM2 transcript in RB tumor tissues from individuals with different rs937283 genotypes was detected by qRT-PCR; Circle, RB patients with AA genotype; Square, RB patients with AG genotype; Triangle, RB patients with GG genotype; *P < 0.05. (**b**) MDM2 and p53 protein levels were evaluated by western blotting; #1 to #8, RB patients with AA genotype (n = 8); #9 to #16, RB patients with AG genotype(n = 8); #17 to #24, RB patients with GG genotype(n = 8). (MDM2, 90 kd; p53, 53 kd; GAPDH, 37 kd) The densitometric analysis of western blotting was shown in Supplementary Fig. S2.

To assess the effect of rs937283 polymorphism on MDM2 expression, we further analyzed mRNA and protein expression levels of MDM2 in RB patients. The effect of rs937283 polymorphism on mRNA expression was evaluated by quantitative real time PCR (qRT-PCR). Similar to transcriptional activity, the results revealed that MDM2 mRNA levels were significantly higher in individuals who carrying AG and GG genotype than those with AA genotype(P < 0.05 for both, Fig. 4a). The differences in the mRNA level between individuals with the AG and GG genotype were also statistically significant(P < 0.05, Fig. 4a). In consistent with the mRNA expression level, the protein expression levels of RB patients carrying the AG or GG genotype were also significantly higher than that of those with AA genotype (Fig. 4b). Conversely, p53 protein expression was significantly lower in RB patients with AA genotype when compared with AG or GG genotype (Fig. 4b). Taken together, the observation of higher MDM2 expression and lower p53 expression in RB patients with GG genotype than those with other genotypes suggested GG genotype at rs937283 as a risk factor for RB.

## Discussion

RB has been the archetypal cancer model that follows the two-hit hypothesis for the initiation of tumor^8^. Although inactivation of the *RB1* tumor suppressor gene seems sufficient for the onset of this tumor, the development of RB is potentially modified by the presence of numerous additional genetic mutations in RB patients^8^,^20^. In the present study, we evaluated the association between rs769412, rs937283 and rs2279744 polymorphisms at MDM2 on RB risk and prognosis. Allele G and genotype GG at rs937283 increased the risk of RB development and were associated with invasion, high tumor aggression. AG + GG at rs937283 was associated with poor prognosis, and was also identified as an independent risk factor for RB prognosis. In addition, our results also showed that the A to G substitution at rs937283 significantly enhanced the transcription activity of the MDM2 gene *in vitro*. Furthermore, we found that MDM2 mRNA and protein were overexpressed *in vivo* in individuals who carried the G allele, suggesting that the MDM2 rs937283 polymorphism is indeed a functional SNP both *in vitro* and *in vivo* as well as a biomarker for risk and prognosis of RB.

During DNA damage response, *TP53* prevents cell proliferation through several mechanisms such as cell cycle arrest and apoptosis. Abnormality in the p53 pathway can yield negative effects on the homeostasis of normal cells; thus, MDM2 can attenuate excess p53 effect as a principal regulator of p53. Specifically, MDM2 can exert its function as an E3 ligase and interact with the terminal transaction domain of *TP53* 21. The production of MDM2 was activated by the cytoplasmic presence of p53 through a negative feedback loop, and as such, both proteins were intricately dependent on each other for proliferation and growth in normal conditions^22^. However, previous *in vitro* studies reported that MDM2 showed p53-independent oncogenic properties that could regulate proliferation, apoptosis, tumor invasion, and metastasis^23^,^24^,^25^. MDM2 protein levels and function were precisely controlled at the transcriptional, translational, and post-translational levels^26^,^27^. Therefore, various SNPs occurring in the MDM2 gene could potentially dysregulate its production.

Until now, the most widely studied MDM2 polymorphism was rs2279744, which is a T > G nucleotide change located in the promoter region of MDM2. rs2279744 can increase the binding affinity of the transcriptional activator SP1 for the MDM2 promoter^13^. This higher affinity potentially leads to increased production of MDM2 mRNA and protein. The increased levels of MDM2, coupled with normal production of p53, may result in lower-than-normal cytoplasmic levels of p53. This in turn can augment DNA damage and hasten cellular transformation^28^. Due to the potential effect of rs2279744 polymorphism on MDM2 production, previous studies have described the role of rs2279744 polymorphism in different neoplasms including lung cancer, cervical cancer, melanoma, and breast cancer^29^,^30^,^31^. However, these studies yielded inconsistent results. Ryan B *et al*. reported that rs2279744 was associated with an increased risk of lung cancer, however, Roszak A *et al*. did not observe any association of rs2279744 with cervical cancer development and clinicopathological features^29^,^30^. Recently, one study conducted by Chen *et al*. also did not observe the significant association of rs2279744 polymorphism with RB risk in a Chinese population^9^. In consistent to Chen’s result, we also could not identify a positive association in Chinese RB patients in the present study. One possibility for this discrepancy was ethnicity. For example, MDM2 rs2279744 polymorphism was not a risk factor for cervical cancer in northeastern Brazilian, Caucasian, or African-American ethnicities, but a stratification-based ethnicity study revealed that the rs2279744 was a significant risk factor for cervical cancer in an Asian population^32^,^33^,^34^. Admittedly, different cancer types may contribute to the difference in association of rs2279744 and tumor risk. Another highly studied MDM2 polymorphism is rs117039649, a G > C nucleotide mutation, which has been reported as a potential antagonist to rs2279744. rs117039649 may override the effect of rs2279744 on SP1-mediated transcription and lead to an overall decrease in MDM2 protein production in the presence of both variant alleles^35^. However, a previous study reported that the rs117039649 SNP in the MDM2 promoter region was not present in Asian populations but was identified in a Caucasian population with an allele frequency of approximately 8% 13. Herein, we also focused on two additional polymorphisms (rs769412 and rs937283) at MDM2 gene and investigated their association with RB risk.

SNPs rs769412 and rs937283 are two novel polymorphisms at MDM2 gene that have been identified in Chinese populations. Recently, Wang *et al*. reported the frequency of MDM2 rs937283 G variant allele was almost 5.8% in a control population, while the GG genotype and G allele were found to have positive correlations with the risk of larynx carcinoma in Chinese Han population^16^. Consistent with that study, our present results revealed the GG genotype and G allele could also increase the risk of RB development with the minor frequency of approximately 7.3%. In Wang’s study, the authors did not discuss the association of this polymorphism with tumor features or tumor prognosis, which we address in our present study. Our result showed positive correlations between rs937283 G allele and tumor invasion and tumor aggression. From the survival analysis, we observed evidence for an independent association between rs937283 G allele with survival, wherein the G allele was associated with an increased risk of poor survival. In tumor, overexpression of MDM2 could substitute for inactivation of p53 in the absence of p53 mutations^36^. Accumulated evidence supported that elevated expression of MDM2 could cause tumorigenesis and was often associated with adverse clinical behaviors of tumors such as invasion, poor outcome, metastasis^37^. Our results proposed that the G > A mutation on rs937283 have ability of increasing MDM2 production and decreasing p53 level. G allele at rs937283 could significantly enhance the transcription activity of MDM2 gene *in vitro. In vivo*, both MDM2 mRNA and protein levels were elevated in RB patients who carried G allele.

It remains controversial concerning the role of rs769412 in tumor development. In Wang’s study, the authors also investigated the association of rs769412 with larynx carcinoma risk^16^. Although no significant association was observed, their results specifically revealed that rs769412 was associated with the significantly increased risk of larynx carcinoma among drinkers. Pine S *et al*. reported no association between rs769412 polymorphism and lung cancer risk^38^, but Rajaraman P *et al*. found rs769412 polymorphism was associated with significantly reduced risk of glioma^39^. In our study, we also did not find a positive association between rs769412 and RB risk. Although we could observe a positive association of rs769412 polymorphism with RB invasion, a small number of individual carrying homozygous GG may limit the accuracy and reliability of this association.

Apparently, there were several limitations in the present study. First, the sample size of the present study was relatively small, which could limit the accuracy and reliability of results. Especially, the specific role of rs769412 in RB required to be further confirmed in a large cohort study due to the low minor allele frequency of rs769412 in Chinese population. Secondly, although we found rs937283 mutation could enhance transcriptional activity of MDM2 gene, the specific mechanism was not clear. One possibility we speculate may be that the A to G substitution of this polymorphism could affect the binding affinity of some transcriptional activator, which will be addressed in future functional studies. Thirdly, we did not evaluated the relationship of these two polymorphisms with other functional MDM2 SNPs such as rs2279744, although previous studies did not observe the differential frequency of rs2279744 polymorphism in RB patients^9^. Forth, the function or power of these two gene variants as a major predictor factor of RB development or RB prognosis in clinical practice was relatively low. Besides, we only conducted this study in Chinese Han population, the frequency of these two polymorphisms in other ethnic groups required to be confirmed. Thus, this study should be replicated in larger independent cohorts of different ethnicities.

In conclusion, our genetic assessment is the first study to evaluate the association of rs769412, rs937283 and rs2279744 with RB. The results showed that rs937283 were associated with the increased risk of RB development. The G allele at rs937283 could increase MDM2 production through enhancing transcriptional activity. In addition, G allele at rs937283 could reflect poor prognosis of RB patients, and may be an independent factor for predicting poor prognosis for RB.

## Methods

### Study Subjects

A total of 137 RB patients recruited from Shandong Provincial Qianfoshan Hospital (Shandong, China) and Yishui Central Hospital (Shandong, China) were enrolled in the present study from September 2009 to August 2012. As controls, 150 gender-matched and ethnicity-matched, unrelated healthy individuals, were also included. All controls were adult individuals who had undergone healthy examination without any history of cancer during the same periods in same hospitals. All aspects of the present study were approved by the Research Ethics Committee of Shandong Provincial Qianfoshan Hospital (NO. QFSYY200908016) and Research Ethics Committee of Yishui Central Hospital (NO. YSCXYY200907003), and all procedures were carried out in accordance with the principles of the Declaration of Helsinki. Written informed consent was obtained from all participants.

### Blood samples and medical data extraction

Approximately 5 ml peripheral vein blood was collected into a tube containing EDTA from each recruited subject. Clinical and laboratory information about all RB patients were extracted from medical records including age at diagnosis, gender, family history of RB, laterality, tumor aggression, tumor invasion, lag time. All 137 RB patients received enucleation therapy and completed 50 months follow-up. Survival time was defined as the time from surgery until the date of RB-related death or last follow-up.

### MDM2 polymorphisms analysis

Genomic DNA was extracted from blood samples using a Genomic DNA kit (Axygen, CA, USA) according to the manufacturer’s protocol and genomic DNA samples were stored at −20 °C until use. Three validated SNPs rs937283, rs2279744 and rs769412 in the MDM2 gene that were previously found to be associated with cancer development were analyzed. Genotyping for these three SNPs was performed using custom TaqMan^®^ SNP Genotyping Assays. Genotyping or allele analysis was carried out with the ABI Prism 7900HT genetic detection system with Taqman^®^ Genotyping Master Mix (ThermoFisher, OK, USA). The final volume of the PCR system was 25 μL including 12.50 ul of Master Mix, 1.25 ul of Assay Mix, 11.25 ul of ddH20, the PCR conditions were as follows: 95 °C for 15 s, 60 °C for 1 min, for 40 cycles. The allelic discrimination plots by automatic allele analysis were shown in Supplementary Fig. S1.

### Construction of reporter plasmids

rs937283 polymorphism was located in the promoter of MDM2 gene, we then determined whether this polymorphism had an effect on gene expression *in vitro*. The MDM2 promoter-luciferase reporter plasmids containing either rs937283 A or G sequence were prepared by amplifying the 300- bp promoter region (from 2010 to 2309) by using primers The primers were 5′-CGG**GGTACC**ATGCTAGTACTGCTACCAG-3′ and 5′-CCG**CTCGAG**ATTCAGTAGCTGTCCTGAC-3′. Two restriction sites including KpnI and XhoI were at 5′-end of forward and reverse primers, respectively. To confirm the matched nucleotides and the plasmid containing either rs937283 A or G allele, the amplified fragments were sequenced. Both the amplified fragments and pGL3-basic vector (Promega, CA, USA) were digested by using the KpnI and XhoI enzymes (NEB, BioLabs lnc, USA). After digestion, the amplified fragments were cloned into pGL3-basic vector and the vectors were sequenced to confirm that there were no errors in the orientation and integrity of each construct.

### Cell culture

Human RB cell lines (Y79, WERI-Rb1) and HeLa cell (Purchased from Institute of Cellular Research, Chinese Academy of Science, Shanghai, China) were cultured in RPMI 1640 containing 10% fetal bovine serum (Hyclone, UT, USA), 100 units/mL penicillin, and 100 ug/mL streptomycin (Gibco, CA, USA). Cells were incubated in a humidified atmosphere of 5% CO2 at 37 °C.

### Luciferase assay

Before transfections, Y79, WERI-Rb1 and HeLa Cells were seeded in 24-well plates. pGL3 luciferase reporter containing rs937283 A or G allele was transfected into cells with the PolyJet DNA *In Vitro* Tranfection Reagent (Signagen Laboratories, MD, USA) according to manufacturer’s instructions. The pGL3-basic vector without an insert was used as a negative control. Renilla plasmid (pRL-SV40) was co-transfected as an internal control. Luciferase assay was performed by using the Dual Glo Luciferase System (Promega, CA, USA). All luciferase activity was normalized against the activity of Renilla luciferase gene. Independent triplicate wells were done for each plasmid.

### RNA isolation and quantitative real-time polymerase chain reaction(qRT-PCR)

Total RNA was isolated from RB tumor tissue using TriZol reagent (Invitrogen, CA, USA), following the manufacturer’s instructions. An ailiquot of the total RNA (1 ug) from each sample was used for cDNA synthesis by a reverse transcription kit (Takara, Japan). After obtaining cDNA, quantitative real-time PCR (qRT-PCR) was performed (20 ul final volume), with qRT-PCR conditions as follows: 98 °C for 10 s, 60 °C for 15 s, 72 °C for 30 s, for 40 cycles. MDM2 mRNA expression was estimated relative to GAPDH using the equation 2^−ΔΔCt^(ΔCt = Ct_MDM2_ − Ct_GAPDH_). The primers were: 5′-GGCAGGGGAGAGTGATACAG-3′ (forward) and 5′-GCCCTCTTCAGCTTGTGTTG-3′ (reverse) for MDM2. And 5′- CCAGAACATCATCCCTGCCT-3′ (forward) and 5′- CCTGCTTCACCACCTTCTTG-3′ (reverse) for GAPDH.

### Protein isolation and western blotting

All 137 RB patients could divided into three groups according to the genotype at rs937283. We randomly selected 8 RB tumor tissues from each groups and then isolated protein. Frozen tumor tissues from RB patients were homogenized on ice and lysed in RIPA buffer containing protease inhibitor (cOmplete, Sigma, CA, USA) and PMSF. The homogenate were sonicated and centrifuged at 12,000 rpm at 4 °C for 5 min to remove cell debris. A BCA assay kit (Beoytime Biotech, Shanghai, China) was used to determine protein concentration and extracted proteins were separated by 8% SDS-PAGE, then separated proteins were transferred onto polyvinylidene difluoride membranes (Millipore, MA, USA). The membrane was blocked with 5% skim milk in TBST (TBS buffer with 0.1% Tween-20), and then incubated with human MDM2 antibody (1:1000 dilution, sc-5304, Mouse Monoclonal IgG, Santa Cruz, USA), p53 antibody (1:1000 dilution, ab28, Mouse Monoclonal IgG, Abcam USA) and GAPDH antibody (1:1000 dilution, sc-32233; Mouse Monoclonal IgG, Santa Cruz, USA) at room temperature for 2 h. Subsequently, horseradish peroxidase(HRP) -conjugated anti-mouse IgG were used as secondary antibody (1:5000 Dilution, Beoytime Biotech, Shanghai, China). Signals were captured by a CCD camera image system (Bio-Rad, CA, USA) with an HRP Chemiluminescent kit (Beoytime Biotech, Shanghai, China).

### Statistical analysis

Continuous data were compared between RB patients and normal controls using Student’s *t* test, while categorical data were compared using Chi-square tests. Association between the genotypes and RB risk were estimated by odds ratios (ORs) using an unconditional logistic regression model. Survival probabilities were estimated by using Kaplan–Meier analysis, and significant differences were analyzed by using the log-rank test. Cox proportional hazards models were used to analyze the associations between genotypes with RB survival. Hazard ratios (HR) and 95% confidence intervals (CI) were estimated using multivariable models. The deviation from Hardy-Weinberg equilibrium was assessed by using χ2 tests. Differences in the expression levels of MDM2 among different genotypes were determined by Student’s t test. All statistical analyses were performed in SPSS 22.0 (SPSS Inc., Chicago, IL) and GraphPad Prism 6.0 (CA, USA). *P* for the two-tailed test less than 0.05 was considered statistically significant.

## Additional Information

**How to cite this article**: Jiao, Y. *et al*. A Functional Polymorphism (rs937283) in the MDM2 Promoter Region is Associated with Poor Prognosis of Retinoblastoma in Chinese Han Population. *Sci. Rep.* **6**, 31240; doi: 10.1038/srep31240 (2016).

## Supplementary Material

Supplementary Information

## Figures and Tables

**Table 1:** Clinical characteristics of retinoblastoma patients and normal controls.

Characteristics	RB patients (n, %)
Gender	
Male	59 (56.9)
Female	78 (43.1)
Age at diagnosis (Months)	
<24	97 (70.8)
>24	40 (29.2)
Family history of RB	
Yes	25 (18.2)
No	112 (81.8)
Laterality	
Unilateral	103 (75.2)
Bilateral	34 (24.8)
Invasion	
Negative	59 (43.1)
Positive	78 (56.9)
Tumor aggression	
Low	58 (42.3)
High	79 (57.7)
Lag-time (Months)	
<3	108 (78.8)
>3	29 (21.2)

Invasion included choroidal invasion or optic nerve invasion.

**Table 2:** Genotype distribution and allele frequencies for rs937283, rs2279744 and rs769412 at MDM2 gene in both RB patients and normal controls.

Genotype		RB Patients (n, %)	Controls (n, %)	HWE	OR	95% CI	*P* value
rs937283	Genotype			0.196			
	A/A	60 (43.8)	91 (60.7)		1	—	
	A/G	59 (43.0)	48 (32.0)		1.86	1.13–3.08	0.02
	G/G	18 (13.2)	11 (7.3)		2.48	1.10–5.62	0.04
	A/G + G/G	77 (56.2)	59 (39.7)		1.98	1.23–3.17	<0.01
	Allele						
	A	179 (65.3)	230 (76.7)		1	—	
	G	95 (34.7)	70 (23.3)		1.74	1.21–2.51	<0.01
rs2279744	Genotype			0.777			
	G/G	41 (29.9)	43 (28.6)		1	—	
	G/T	59 (43.1)	73 (48.7)		0.85	0.49–1.47	0.326
	T/T	37 (27.0)	34 (22.7)		1.14	0.61–2.15	0.402
	T/G + T/T	96 (70.1)	107 (71.4)		0.94	0.57–1.57	0.458
	Allele						
	G	141 (51.5)	159 (53.0)		1	—	
	T	133 (48.5)	141 (47.0)		1.06	0.77–1.48	0.388
rs769412	Genotype			0.100			
	A/A	98 (71.5)	115 (76.7)		1	—	
	A/G	30 (21.9)	30 (20.0)		1.13	0.73–1.77	0.344
	G/G	9 (6.6)	5 (3.3)		2.02	0.70–5.84	0.147
	A/G + G/G	39 (28.5)	35 (23.3)		1.34	0.89–2.02	0.102
	Allele						
	A	226 (82.5)	260 (86.7)		1	—	
	G	48 (17.5)	40 (13.3)		1.314	0.89–1.93	0.101

**Table 3:** The association of rs937283, rs2279744 and rs769412 with clinicopathological characteristics in RB patents.

Characteristics	rs937283					rs2279744					rs769412				
	Genotype			Allele		Genotype			Allele		Genotype			Allele	
	A/A	A/G	G/G	A	G	G/G	G/T	T/T	G	T	A/A	A/G	G/G	A	G
Gender															
Female	37	31	10	105	51	26	32	20	84	72	56	18	4	130	26
Male	23	28	8	74	44	15	27	17	57	61	42	12	5	96	22
OR (95%)	1.0 (−)	1.45 (0.70–3.01)	1.29 (0.44–3.74)	1.0 (−)	1.22 (0.74–2.02)	1.0 (−)	1.46 (0.65–3.31)	0.99 (0.44–2.27)	1.0 (−)	1.25 (0.77–2.02)	1.0 (−)	0.89 (0.39–2.04)	1.68 (0.42–6.59)	1.0 (−)	1.15 (0.61–2.14)
*P* value	—	0.357	0.785	—	0.44	—	0.24	0.576	—	0.216	—	0.48	0.35	—	0.75
Age at diagnosis															
<24 months	44	40	13	128	66	28	44	25	100	94	70	21	6	161	33
>24 months	16	19	5	51	29	13	15	12	41	39	28	9	3	65	15
OR (95%)	1.0 (−)	1.31 (0.59–2.88)	1.06 (0.33–3.44)	1.0 (−)	1.10 (0.64–1.90)	1.0 (−)	0.73 (0.30–1.77)	1.03 (0.40–2.68)	1.0 (−)	1.01 (0.60–1.70)	1.0 (−)	1.07 (0.44–2.62)	1.25 (0.29–5.35)	1.0 (−)	1.13 (0.57–2.21)
*P* value	—	0.55	0.573	—	0.78	—	0.321	0.569	—	0.535	—	0.52	0.72	—	0.73
Family history															
Yes	12	10	3	34	16	6	11	8	23	27	17	6	2	40	10
No	48	49	15	145	79	35	48	29	118	106	81	24	7	186	38
OR (95%)	1.0 (−)	1.23 (0.4–3.10)	1.25 (0.31–5.03)	1.0 (−)	1.16 (0.60–2.23)	1.0 (−)	0.75 (0.25–2.22)	0.62 (0.19–2.00)	1.0 (−)	0.77 (0.41–1.42)	1.0 (−)	0.84 (0.30–2.37)	0.74 (0.14–3.89)	1.0 (−)	0.87 (0.38–1.78)
*P* value	—	0.814	0.526		0.74	—	0.404	0.306		0.243	—	0.79	0.66		0.68
Laterality															
Unilateral	45	45	13	135	71	31	47	25	109	97	77	19	7	173	33
Bilateral	15	14	5	44	24	10	12	12	32	36	21	11	2	53	15
OR (95%)	1.0 (−)	0.93 (0.40–2.16)	1.15 (0.35–3.78)	1.0 (−)	1.04 (0.58–1.84)	1.0 (−)	0.79 (0.30–2.06)	1.49 (0.55–4.01)	1.0 (−)	1.26 (0.73–2.19)	1.0 (−)	2.12 (0.88–5.15)	1.05 (0.20–5.42)	1.0 (−)	1.48 (0.75–2.94)
*P* value	—	0.521	0.77		0.51	—	0.404	0.296		0.243	—	0.10	0.62		0.27
Invasion															
Negative	35	20	4	90	28	16	27	16	59	59	49	8	2	106	12
Positive	25	39	14	89	67	25	32	21	82	74	49	22	7	120	36
OR (95%)	1.0 (−)	**2.73** (**1.30**–**5.75**)	**4.90** (**1.44**–**16.6**)	1.0 (−)	**2.42** (**1.43**–**4.11**)	1.0 (−)	0.76 (0.34–1.71)	0.84 (0.34–2.07)	1.0 (−)	0.90 (0.56–1.46)	1.0 (−)	**2.75** (**1.12**–**6.77**)	3.50 (0.69–17.7)	1.0 (−)	**2.65** (**1.31**–**5.36**)
*P* value	—	0.01	0.01		<0.01	—	0.322	0.441		0.383	—	0.03	0.65		<0.01
Aggression															
Low	34	19	5	87	29	18	21	19	57	59	43	12	3	98	18
High	26	40	13	92	66	23	38	18	84	74	55	18	6	128	30
OR (95%)	1.0 (−)	**2.75** (**1.30**–**5.81**)	**3.40** (**1.08**–**10.7**)	1.0 (−)	**2.15** (**1.27**–**3.64**)	1.0 (−)	1.42 (0.63–3.20)	0.74 (0.30–1.81)	1.0 (−)	0.85 (0.53–1.38)	1.0 (−)	1.18 (0.51–2.70)	1.56 (0.37–6.61)	1.0 (−)	1.28 (0.67–2.42)
*P* value	—	0.01	0.04		0.53	—	0.264	0.333		0.296	—	0.03	0.31		0.04
Lag-time															
<3 months	49	45	14	143	73	33	47	28	113	103	78	24	6	180	36
>3 months	11	14	4	36	22	8	12	9	28	30	20	6	3	46	12
OR (95%)	1.0 (−)	1.39 (0.57–3.37)	1.27 (0.35–4.6)	1.0 (−)	1.20 (0.66–2.18)	1.0 (−)	1.05 (0.39–2.86)	1.33 (0.45–3.89)	1.0 (−)	1.18 (0.66–2.10)	1.0 (−)	0.98 (0.35–2.71)	1.95 (0.45–8.48)	1.0 (−)	1.30 (0.63–2.70)
*P* value	—	0.51	0.74	—	0.64	—	0.564	0.405	—	0.345	—	0.59	0.40	—	0.56

**Table 4:** Multivariate Cox proportional hazard analysis of prognostic factors for overall survival rates of RB patients.

Factors	Categories	Multivariate	
		HR (95% CI)	*p*
Age	>24 m/<24 m	0.86(0.49–2.02)	0.42
Gender	Male/Female	1.07(0.82–2.49)	0.56
Family history of RB	Yes/No	1.24(0.53–2.09)	0.27
Laterality	Unilateral/Bilateral	0.66(0.32–1.14)	0.09
Tumor aggression	Low/High	2.33(0.81–2.46)	0.13
Lag-time	>3 m/<3 m	3.17(1.28–4.66)	<0.05
Invasion	Yes/No	3.87(1.72–5.52)	<0.05
Genotype at rs2279744	GT + TT/GG	2.03(0.37–3.33)	0.48
Genotype at rs769412	AG + GG/AA	1.56 (0.45–2.99)	0.35
Genotype at rs937283	AG + GG/AA	2.01 (1.04–3.95)	<0.05

HR: Hazard Ratio; 95% CI: 95% Confidence Interval; m, months.
