# Supplementary material for: A Functional Polymorphism (rs937283) in the MDM2 Promoter Region is Associated with Poor Prognosis of Retinoblastoma in Chinese Han Population
Source: Sci Rep. 2016 Aug 10;6:31240. doi: 10.1038/srep31240 (PMC4979029; doi:10.1038/srep31240)

Title: A Functional Polymorphism (rs937283) in the MDM2 Promoter Region is Associated with Poor Prognosis of Retinoblastoma in Chinese Han Population

Author: Yongfa Jiao, Zhongming Jiang, Yuxia Wu, Xiaochong Chen, Xing Xiao, Haiying Yu

Corresponding author: Haiying Yu, E-mail: yuhyradiol@163.com

Supplementary Figure S1: MDM2 polymorphism analysis; Taqman SNP genotyping plots of rs769412 (Left), rs937283 (Middel), rs2279744 (Right) in MDM2 gene. The vertical axis indicated a wild-type (WT) AA homozygote (for rs769412 and rs937283) or GG homozygote (rs2279744), the horizontal axis indicated mutant type GG homozygote (rs769412 and rs937283) or TT homozygote (rs2279744), and the diagonal was AG heterozygote or GT heterozygote (rs2279744). Blue circle, AA or GG genotype; Green Circle, AG or GT genotype; Red Circle, GG or TT genotype.

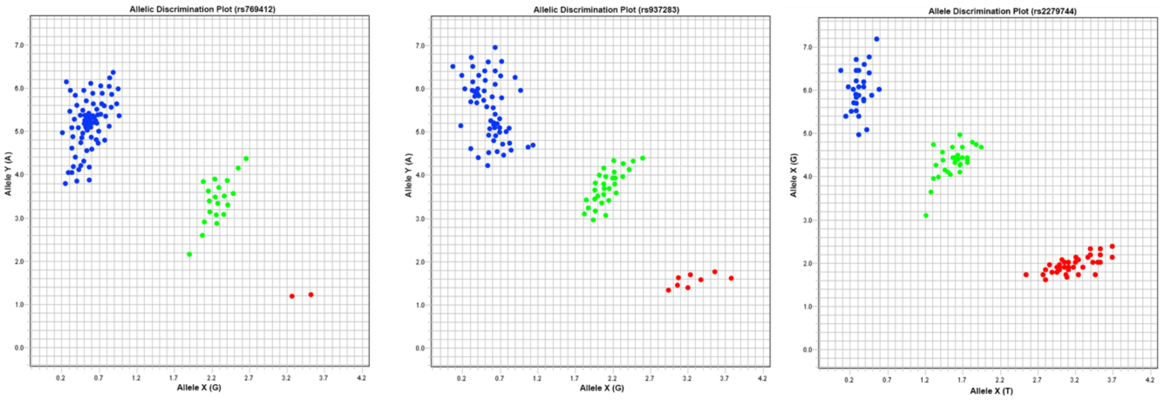

Supplementary Figure S2: Densitometric analysis of Western blotting of Figure 4b. Data represent the mean ±SD of 3 separate experiments. *P<0.01 between AA genotype and AG, GG genotypes.

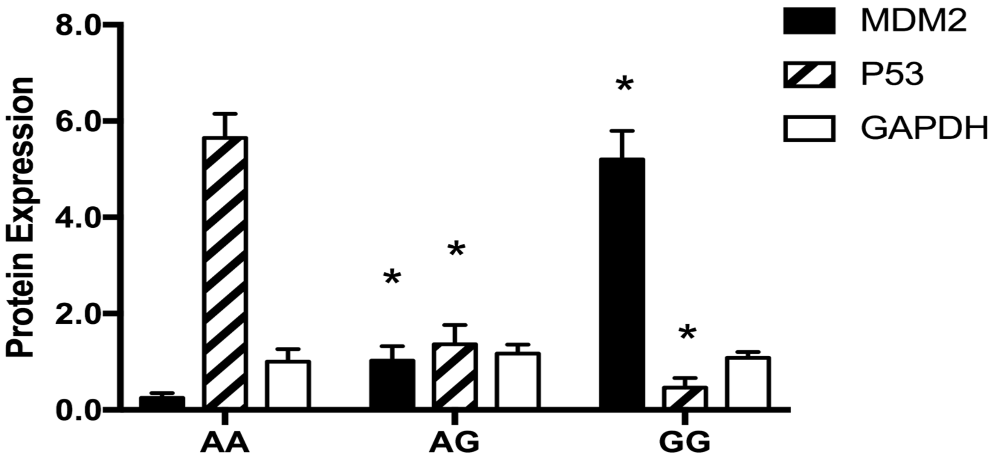

Supplement: Supplementary Information [file srep31240-s1.pdf]
